# The Impact of Ovariectomy on Calcium Homeostasis and Myofilament Calcium Sensitivity in the Aging Mouse Heart

**DOI:** 10.1371/journal.pone.0074719

**Published:** 2013-09-18

**Authors:** Elias Fares, W. Glen Pyle, Gibanananda Ray, Robert A. Rose, Eileen M. Denovan-Wright, Robert P. Chen, Susan E. Howlett

**Affiliations:** 1 Department of Pharmacology, Dalhousie University, Halifax, Nova Scotia, Canada; 2 Department of Physiology & Biophysics, Dalhousie University, Halifax, Nova Scotia, Canada; 3 Department of Medicine (Geriatric Medicine), Dalhousie University, Halifax, Nova Scotia, Canada; 4 Cardiovascular Research Group, Department of Biomedical Sciences, University of Guelph, Guelph, Ontario, Canada; 5 Pediatric Cardiology, IWK Health Centre and Dalhousie University, Halifax, Nova Scotia, Canada; University of Canberra, Australia

## Abstract

This study determined whether deficiency of ovarian estrogen starting very early in life promoted age-associated Ca^2+^ dysregulation and contractile dysfunction in isolated ventricular myocytes. Myocytes were isolated from anesthetized C57BL/6 female mice. Animals received an ovariectomy or sham-operation at one month and were aged to ~24 months. Excitation-contraction coupling parameters were compared in fura-2 loaded myocytes (37°C). While Ca^2+^ transients were larger and faster in field-stimulated myocytes from ovariectomized mice, ovariectomy had no effect on peak fractional shortening. Similarly, ovariectomy had no effect on fractional shortening measured *in vivo* by echocardiography (values were 60.5 ± 2.9 vs. 60.3 ± 2.5% in sham and ovariectomized, respectively; n=5 mice/group). Ovariectomy did decrease myofilament Ca^2+^ sensitivity, as evidenced by a 26% increase in the Ca^2+^ required to activate actomyosin MgATPase in ovariectomized hearts. Larger Ca^2+^ transients were attributable to a 48% increase in peak Ca^2+^ current, along with an increase in the amplitude, width and frequency of Ca^2+^ sparks measured in fluo-4 loaded myocytes. These changes in Ca^2+^ handling were not due to increased expression of Ca^2+^ channels (Ca_v_1.2), sarcoplasmic reticulum Ca^2+^ ATPase (SERCA2) or Na^+^-Ca^2+^ exchanger in ovariectomized hearts. However, ovariectomy increased sarcoplasmic reticulum Ca^2+^ stores by ~90% and promoted spontaneous Ca^2+^ release from the sarcoplasmic reticulum when compared to sham controls. These observations demonstrate that long-term ovariectomy promotes intracellular Ca^2+^ dysregulation, reduces myofilament Ca^2+^ sensitivity and increases spontaneous Ca^2+^ release in the aging female heart.

## Introduction

Studies in humans have shown that cardiac contractile function declines with age, even in the absence of overt cardiovascular disease [1,2]. Although contractile function is relatively well preserved at rest, the ability to increase contractile force under conditions of higher demand, such as exercise, declines with age [1]. Studies in intact hearts and cardiac tissues from aged rats also show that the ability to augment force in response to positive inotropic stimuli is compromised in the aging heart [2]. This age-related decrease in cardiac contractile function is due, at least in part, to a decrease in the ability of individual ventricular myocytes to contract [3-6].

Most previous studies of the impact of age on cardiac contractile function in animal models have used hearts, tissues and myocytes from male animals. However, there is evidence that the effect of age on cardiac contractile function differs between the sexes. Studies have shown that contractile force, fractional shortening and left ventricular function deteriorate with age in male rats and non-human primates, but are unaffected by age in female animals [7-9]. Previous work from our laboratory and others has shown that the ability of individual ventricular myocytes to contract declines with age in male but not female rats and mice [9-11]. This arises as a consequence of a reduction in the magnitude of the Ca^2+^ transient required to initiate contraction [9-11]. These findings suggest that sex differences in cardiac contractility in the aging heart may be linked to effects of sex steroid hormones on myocardial Ca^2+^ handling. However, little is known about the influence of sex steroid hormones such as estrogen on cardiac Ca^2+^ homeostasis in the setting of aging.

Cardiac myocytes possess estrogen receptors [12] and evidence suggests that chronic exposure to estrogen modifies intracellular Ca^2+^ homeostasis. Studies have shown that Ca^2+^ transients and contractions are smaller and slower in ventricular myocytes from young adult female rats when compared to age-matched males [9,13,14]. However, bilateral ovariectomy (OVX) of young adult females increases the speed and magnitude of Ca^2+^ transients and contractions compared to sham-operated controls [15-19] but cf. [20,21]. We have shown that this is not due to an increase in Ca^2+^ current, but arises from increased sarcoplasmic reticulum (SR) Ca^2+^ release as a consequence of increased SR stores and larger Ca^2+^ sparks [15]. These findings suggest that removal of ovarian estrogen in young adult females enhances SR Ca^2+^ release and leads to Ca^2+^ transients and contractions that are similar to those seen in myocytes from young adult males. It is possible that long term estrogen deprivation, starting early in life, may lead to age-associated Ca^2+^ dysregulation and contractile dysfunction as seen in myocytes from aged males. However, whether long-term OVX alters Ca^2+^ homeostasis and causes deterioration in cardiac contractile function in the aging female heart has not been investigated.

The overall aim of this study was to determine whether long-term OVX modifies myocardial Ca^2+^ homeostasis and disrupts contractile function in the aging mouse heart. Studies used very old (e.g. ~24 month old) female C57BL6/J mice that received either a bilateral OVX or sham surgery at an early age (e.g. one month of age). Ventricular myocytes were loaded with Ca^2+^-sensitive fluorophores to investigate specific Ca^2+^ handling mechanisms. Contractions, Ca^2+^ transients, Ca^2+^ currents, sarcoplasmic reticulum (SR) Ca^2+^ content and Ca^2+^ sparks were compared in myocytes from aged sham and OVX mice. *In vivo* cardiac function was evaluated with echocardiography and myofilament Ca^2+^ sensitivity was assessed by measurement of actomyosin MgATPase activity. Ca^2+^ handling proteins were assessed by Western blot analysis. Our results showed that long-term OVX reduced myofilament Ca^2+^ sensitivity, promoted cardiomyocyte Ca^2+^ dysregulation and increased spontaneous SR Ca^2+^ release in the aging female heart. 

## Materials and Methods

For full details of Methods, please refer to Methods S1 online.

### Ethics Statement

Protocols were approved by the Dalhousie University Committee on Laboratory Animals (No. 12-022) and followed Canadian Council on Animal Care Guide to the Care and Use of Experimental Animals (CCAC, Ottawa, ON: Vol 1, 2nd edition, 1993: Vol. 2, 1984). Sodium pentobarbital anesthesia was used and all efforts were made to reduce suffering.

### Myocyte studies

Myocytes were isolated from 20-24 mos female C57BL/6 mice that had either sham surgery or OVX at 1 mos. Ventricular myocytes were isolated by enzymatic digestion as described in detail previously [11]. OVX was confirmed by uterine atrophy. All experiments were conducted at 37°C. Contractions (unloaded cell shortening), transmembrane currents and Ca^2+^ transients (fura-2 AM) were measured simultaneously as described in our previous studies [22-25]. In voltage clamp experiments, membrane potentials and transmembrane currents were recorded with an Axoclamp 2B amplifier (switch clamp, 5-8 kHz). Transient outward K^+^ current was inhibited by 4-aminopyridine (4 mM) and steps were made from -40 mV to inactivate Na^+^ current. SR Ca^2+^ load was measured by rapid application of 10 mM caffeine in 0 Na^+^/0 Ca^2+^ buffer to inhibit Ca^2+^ extrusion via Na^+^-Ca^2+^ exchange [26,27]. Action potentials were measured in separate experiments, in cells not loaded with fura-2. In field stimulation experiments, cells were paced with platinum electrodes. Ca^2+^ sparks were measured in fluo-4 AM-loaded myocytes as described previously [14] and analyzed with SparkMaster [28].

### Echocardiography

Two-dimensional guided M-mode echocardiography was performed in anesthetized mice (2% isoflurane). ECG electrodes were placed subcutaneously and mice were assessed with a high-resolution linear transducer connected to a Vivid 7 imaging system.

### Myofilament studies

Myofilaments were isolated from the ventricles and frozen until use as described previously [29]. Actomyosin MgATPase activity was determined with techniques that have been previously described [29]. Myofilaments (25 mg) were incubated in activating solutions containing varying levels of free Ca^2+^ as described earlier [30].

### Western blots

Ventricles were homogenized (60 s) in buffer (5 mM EDTA, 20 mM HEPES, 2% LDS, 10% glycerol, 1mM AEBSF, 800 nM apoprotin, 20 µM leupeptin, 10 µM pepstatin, 50 µM bestatin, 15 µM E-64). Tissues were sonicated 3 times and flash frozen in liquid nitrogen after each sonication step. Samples were then heated (5 mins, 70°C), cooled on ice (5 mins), centrifuged (12,000 rpm, 8 mins, 4°C) and the supernatant was stored (-80°C). Protein concentration was determined with a DC Protein Assay (BioRad). Antibodies used were rabbit anti- Ca_v_1.2 polyclonal antibody (Alomone, ACC-003-AG; 1:2000), mouse anti-NCX monoclonal antibody (SWANT, R3F1; 1:1000), mouse anti-SERCA2 monoclonal antibody (Affinity Bioreagents, MA3-919; 1:2000) and rabbit anti-Na/KATPase polyclonal antibody (Abcam; 1:2000). Secondary antibodies were goat anti-mouse HRP-conjugated polyclonal antibody (Abcam, 1:30,000) and goat anti-rabbit HRP-conjugated polyclonal antibody (Abcam, 1:30,000). Protein was resolved on an 8% polyacrylamide gel, transferred to nitrocellulose membrane and blocked. Loading controls were anti-Na/K ATPase antibody (Ca_v_1.2 and NCX) or amido black (SERCA2). Signals were visualized with Immuno-Star™ WesternC™ Kit (BioRad) and quantified with Quantity One software.

### Statistics

Statistical analyses were performed with SigmaStat (version 3.1, Systat Software, Inc.). Differences between means were evaluated by a t-test or two-way repeated-measures analysis of variance. Graphs were constructed with Sigmaplot (version 8.0, Systat Software, Inc). Data are presented as means ± SEM and differences were considered significant if p<0.05. 

## Results

### Physical characteristics of sham and OVX mice

Selected physical characteristics of the sham and OVX mice used in this study were compared as shown in Table 1. Sham and OVX mice were the same age (24.5 ± 0.5 vs. 23.2 ± 0.5 months for sham and OVX mice, respectively; n=22 mice per group) and had similar body weights (Table 1). Ventricle weight and ventricle-to-body weight ratios were also similar in the two groups (Table 1), while uterine dry weights were significantly reduced in OVX mice (Table 1). These findings show that while sham and OVX aged females were similar in weight and had similar heart sizes, OVX caused marked uterine atrophy.

**Table 1:** Selected Physical Characteristics of the Animals and Myocytes Used In This Study.

**Parameter**	**Sham (n**)	**OVX (n**)	**p value**
Body weight (g)	32.2 ± 1.7 (18)	36.7 ± 2.4 (15)	p=0.130
Ventricle weight (mg)	181.4 ± 12.9 (5)	178.5 ± 9.8 (4)	p=0.869
Ventricle weight/body weight (mg/g)	6.3 ± 0.4 (5)	5.2 ± 0.3 (4)	p=0.119
Uterine dry weight (mg)	19.6 ± 2.0 (13)	5.3 ± 0.7* (16)	P<0.001
Myocyte capacitance (pF)	302.6 ± 20.6 (15)	230.7 ± 16.3* (16)	p=0.010
Myocyte volume (pL)	35.4 ± 1.6 (15)	29.8 ± 1.3* (16)	p=0.010

Numbers represent mean ± SEM; values in brackets represent the number of replicates. The * denotes significantly different from sham values, p<0.05.

To determine whether OVX affected cardiomyocyte size, myocyte capacitance and myocyte volume were compared in sham and OVX animals. Cell capacitance was 24% lower in OVX myocytes compared to sham cells (Table 1). Cell volume, calculated from cell capacitance as described previously [31], also was reduced in myocytes from OVX mice when compared to sham cells (Table 1). These findings demonstrate that cardiomyocyte volume and membrane area were reduced by long-term OVX in the aging female heart.

### OVX modifies Ca^2+^ homeostasis and contractions in field-stimulated cardiomyocytes

**Figure 1:**
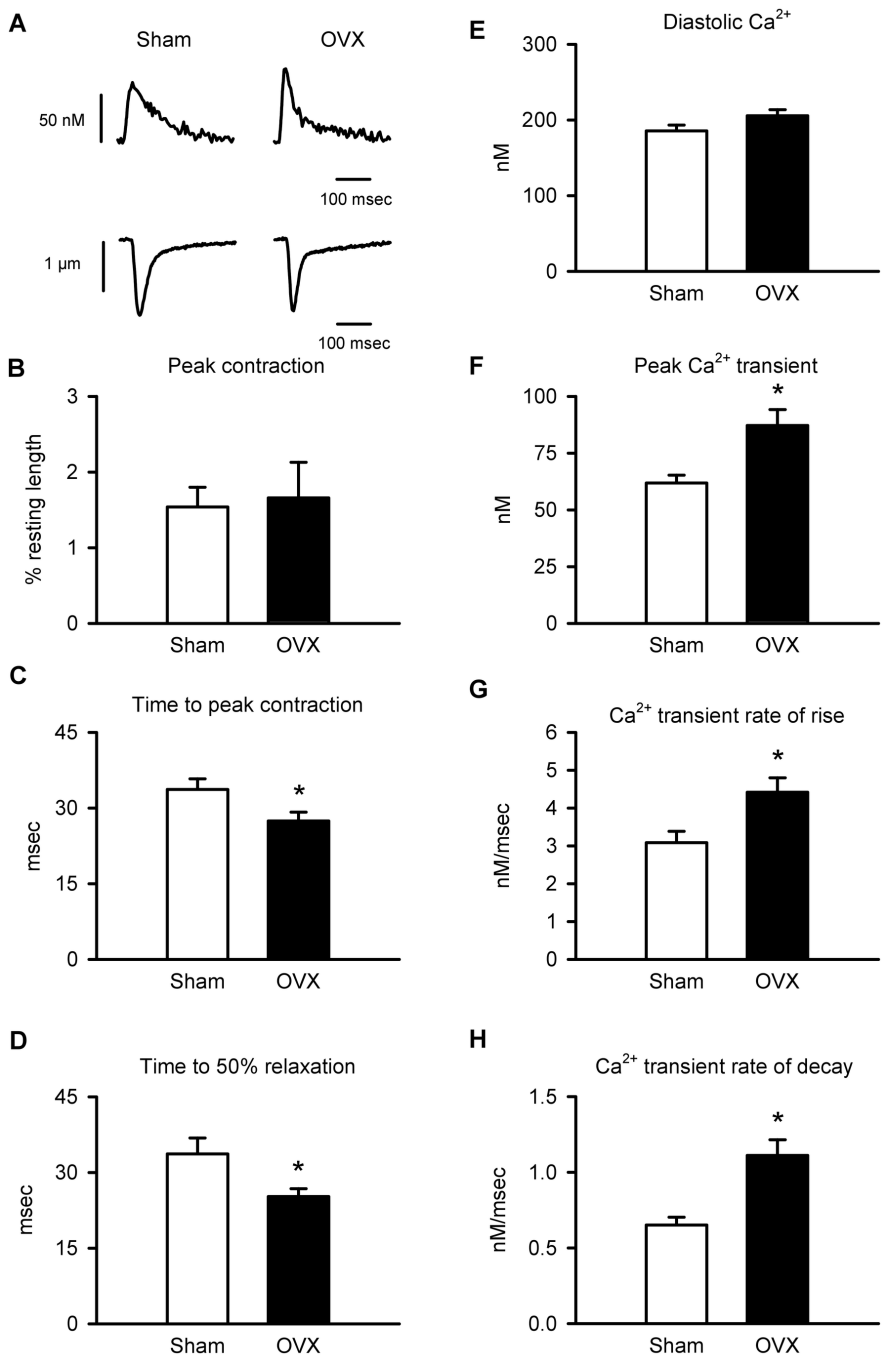
Ca^2+^ transients were larger and faster in field-stimulated myocytes from OVX mice compared to controls. **A**. Ca^2+^ transients (top) and contractions (bottom) recorded in myocytes from sham and OVX mice (2 Hz). **B**. Peak contraction, normalized to resting cell length, was similar in sham and OVX myocytes. **C**,**D**. Mean time-to-peak and time-to-50% relaxation of contraction were reduced by OVX. **E**. Diastolic Ca^2+^ levels were similar in the two groups. **F**. Peak Ca^2+^ transients were larger in OVX cells than sham controls. **G**,**H**. Mean rate-of-rise and average rate of decay of the Ca^2+^ transient were both increased by OVX (n=25 sham and 26 OVX myocytes; *p<0.05; t-test).

To determine whether long-term OVX influenced Ca^2+^ homeostasis and contractile function, contractions and Ca^2+^ transients were simultaneously recorded in sham and OVX myocytes that were field-stimulated at 2 Hz. Figure 1A shows examples of Ca^2+^ transients and contractions recorded from sham and OVX myocytes paced at 2 Hz. Mean peak contractions, normalized to cell resting length, did not differ between sham and OVX cells (Figure 1B). However, time-to-peak contraction (Figure 1C) and time-to-50% relaxation (Figure 1D) were reduced by OVX. Intracellular Ca^2+^ concentrations also were compared in sham and OVX myocytes. Mean diastolic Ca^2+^ levels were similar in both groups (Figure 1E), but Ca^2+^ transients were 41% larger in OVX cells than in sham controls (Figure 1F). In addition, the average rate of rise of the Ca^2+^ transient (Figure 1G) was increased by 43% in OVX myocytes. The rate of decay (Figure 1H) also was increased by 71% in cells from OVX mice. To determine whether these differences in contractions and Ca^2+^ transients were present when cells were challenged with more physiological pacing rates, we conducted similar studies in cells paced at 8 Hz (Table 2). As in cells paced at 2 Hz, contractions were similar in magnitude but had a faster time course in OVX cells compared to sham controls (Table 2). In addition, Ca^2+^ transients were larger and had a more rapid time course in OVX cells compared to sham controls (Table 2), as we observed when cells were paced at 2 Hz. The only difference between results obtained at these two pacing frequencies is that the resting levels of diastolic calcium were significantly higher in OVX cells compared to sham controls at 8 Hz, whereas this was only a trend in the 2 Hz data (Table 2). Together, these observations show that Ca^2+^ transients were larger and faster in myocytes from aged OVX females when compared to sham controls. However, despite the marked increase in peak Ca^2+^ transients, OVX had no effect on peak contraction in cardiomyocytes. The mechanistic basis for this was explored.

**Table 2:** Responses Myocytes Paced at a Frequency of 8 Hz.

**Parameter**	**Sham (n**)	**OVX (n**)	**p value**
Fractional shortening (%)	3.13 ± 0.5 (17)	3.28 ± 0.3 (23)	p=0.795
Time-to-peak contraction (msec)	34.9 ± 1.2 (17)	29.5 ± 1.1* (23)	p=0.004
Time-to-50% relaxation (msec)	27.3 ± 1.5 (17)	21.1 ± 0.9* (23)	p=0.001
Ca^2+^ transient (nM)	67.3 ± 4.8 (14)	92.4 ± 6.2* (22)	p=0.007
Diastolic Ca^2+^ (nM)	246.6 ± 11.7 (14)	284.8 ± 10.7* (22)	p=0.025
Rate of Ca^2+^ transient rise(nM/msec)	3.60 ± 0.33 (14)	5.18 ± 0.33* (22)	p=0.002
Velocity to 50% transient decay (nM/msec)	0.96 ± 0.08 (14)	1.53 ± 0.10* (22)	p=0.001

Numbers represent mean ± SEM; values in brackets represent the number of cells. The * denotes significantly different from sham values, p<0.05.

### In vivo cardiac contractile function

**Figure 2:**
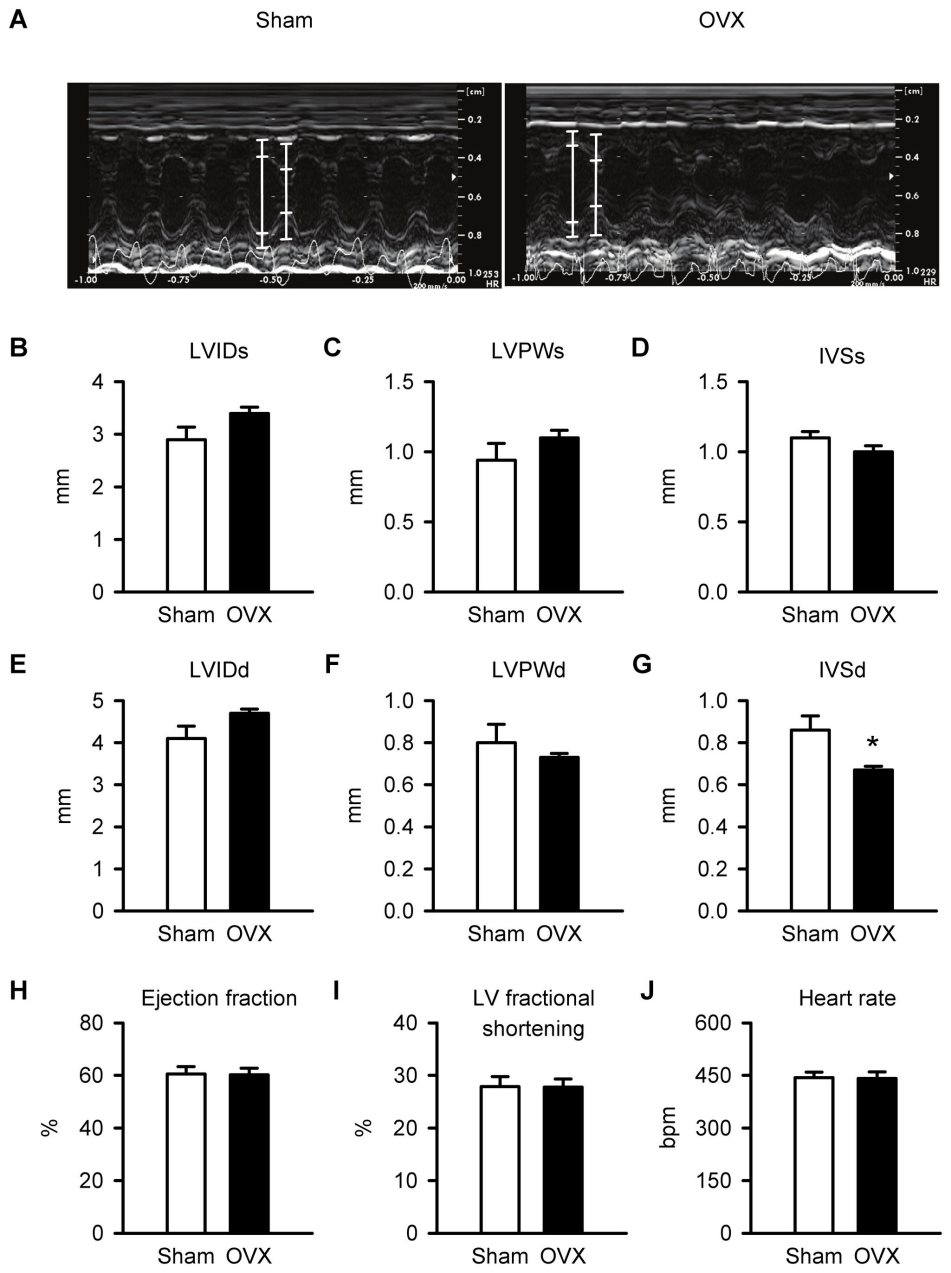
*In vivo* ventricular structure, function and heart rate were similar in sham and OVX mice. **A**. M-mode images of cardiac function from sham and OVX mice. The white lines in each image denote the lumen edges in systole and diastole. **B**,**C**,**D**. Left ventricular internal diameter in systole (LVIDs), left ventricular posterior wall thickness in systole (LVPWs) and interventricular septal thickness in systole (IVSs) were similar in sham and OVX hearts. **E**,**F**. Left ventricular internal diameter in diastole (LVIDd) and left ventricular posterior wall thickness in diastole (LVPWd) were unaffected by OVX. **G**. By contrast, interventricular septal thickness in diastole (IVSd) was reduced significantly by OVX. **H**,**I**,**J**. Mean values for ejection fraction, left ventricular fractional shortening and heart rate were identical in sham and OVX mice (n=6 sham and 6 OVX mice; *p<0.05; t-test).

To determine whether *in vivo* contractile function was affected by long-term OVX, two-dimensional guided M-Mode echocardiography was performed. Figure 2A illustrates representative M-mode recordings from sham and OVX mice. Mean results showed all structural parameters measured in systole (e.g. LVIDs, LVPWs and IVSs) were similar in sham and OVX hearts (Figure 2B-D). Although most structural parameters measured in diastole (e.g. LVIDd and LVPWd) were similar in the two groups (Figure 2E,F), IVSd was smaller in OVX mice when compared to sham controls (Figure 2G). Thus, there were few differences in structure between sham and OVX hearts. However, mean values for ejection fraction, left ventricular fractional shortening and heart rate also were not affected by long term OVX (Figure 2H-J). These measurements showed that *in vivo* ventricular function was comparable in aged sham and OVX mice.

### Myofilament Ca^2+^ sensitivity is reduced by long-term OVX

**Figure 3:**
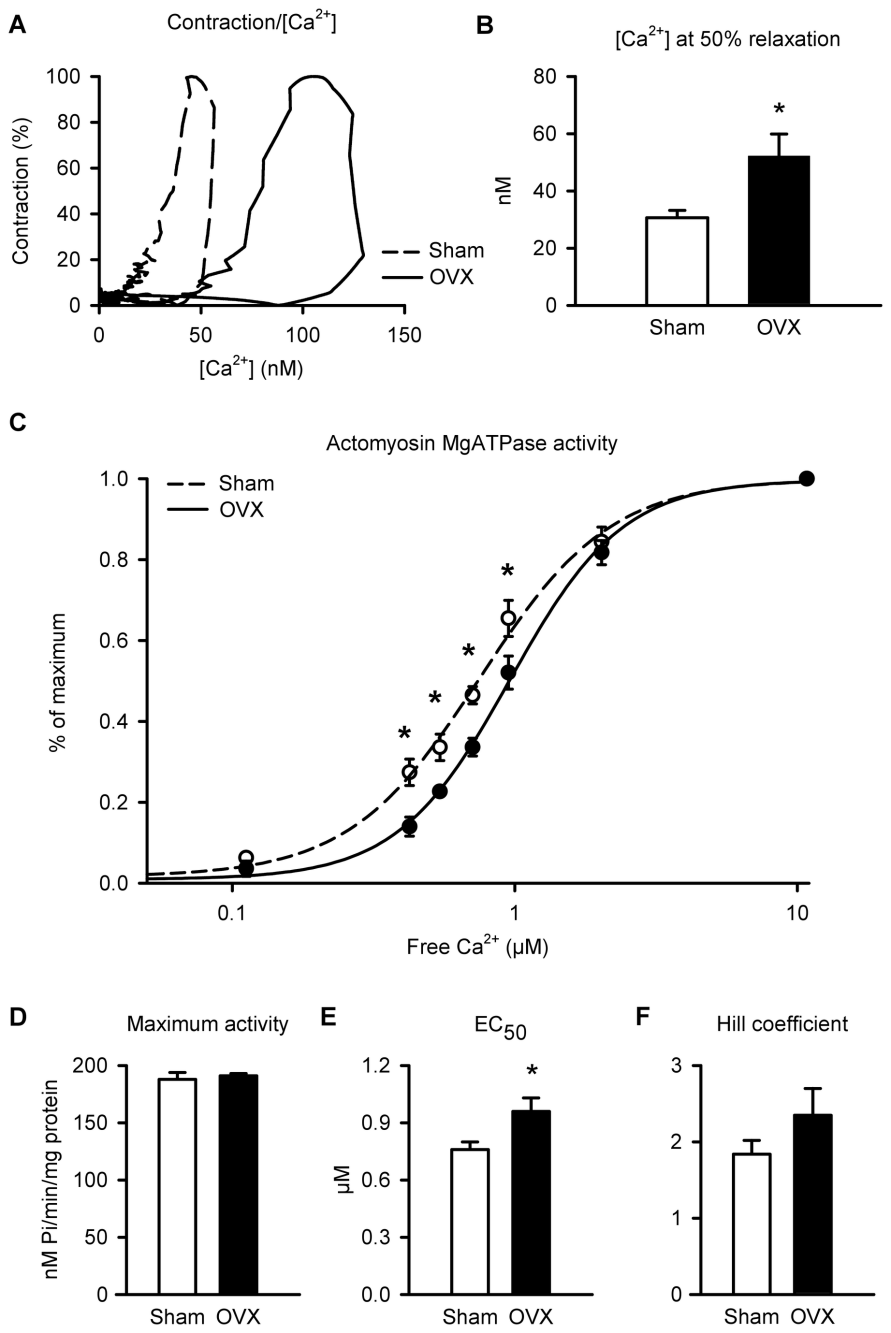
Ca^2+^ sensitivity of the myofilaments was reduced by OVX. **A**. Shortening-[Ca^2+^] phase loop plots show that OVX caused a rightward shift in the descending phase of the loop, consistent with a decrease in myofilament Ca^2+^ sensitivity. **B**. Ca^2+^ levels at 50% relaxation, measured to quantify this shift, were higher in OVX myocytes than in sham controls. **C**. Myofilament Ca^2+^ sensitivity was lower in OVX hearts, as indicated by a rightward shift in the actomyosin MgATPase activity-[Ca^2+^] curves. **D**. Maximum actomyosin MgATPase activity was similar in both groups. **E**,**F**. OVX increased mean EC_50_ values compared to sham hearts but had no effect on the Hill coefficient (n=25 sham and 26 OVX myocytes for panel B; n=5 sham and 4 OVX hearts for actomyosin MgATPase assay; *p<0.05; Panel C was analyzed with a two-way ANOVA; main factors of free Ca^2+^ and OVX were significant; other data were analyzed with a t-test; *denotes significant differences).

As OVX had no effect on cardiomyocyte contraction even though Ca^2+^ transients were enhanced, it is possible that OVX reduced myofilament Ca^2+^ sensitivity. To evaluate myofilament Ca^2+^ sensitivity, phase-loop plots of individual shortening-[Ca^2+^] relationships were compared in sham and OVX cells (Figure 3A); the descending portion of the loop provides a dynamic index of myofilament Ca^2+^ sensitivity [32,33]. Representative plots show that OVX shifted the descending portion of the loop to the right compared to sham controls (Figure 3A). This shift was quantified by comparing the Ca^2+^ concentration at 50% relaxation, as in previous studies [32]. Ca^2+^ levels at 50% relaxation were increased by 46% in OVX myocytes compared to sham controls (Figure 3B). These data are consistent with a decrease in myofilament Ca^2+^ sensitivity in the aged OVX group.

Next, myofilament Ca^2+^ sensitivity was assessed directly with a Carter assay to measure actomyosin MgATPase activity. Figure 3C shows that the actomyosin MgATPase activity-Ca^2+^ curve was shifted to the right by OVX, when compared to sham controls. Maximal actomyosin MgATPase activity at saturating levels of free calcium (~10 mM) was not different between the two groups (Figure 3D). However, the average concentration of Ca^2+^ required to produce half the maximum response (EC_50_ values) increased by 26% in OVX when compared to sham controls (Figure 3E). Cooperativity, measured by the Hill coefficient, did not differ significantly between the two groups (Figure 3F). These results indicate that long-term OVX reduced myofilament Ca^2+^ sensitivity in the aging female heart.

### EC-coupling mechanisms are disrupted in cardiomyocytes from OVX mice

**Figure 4:**
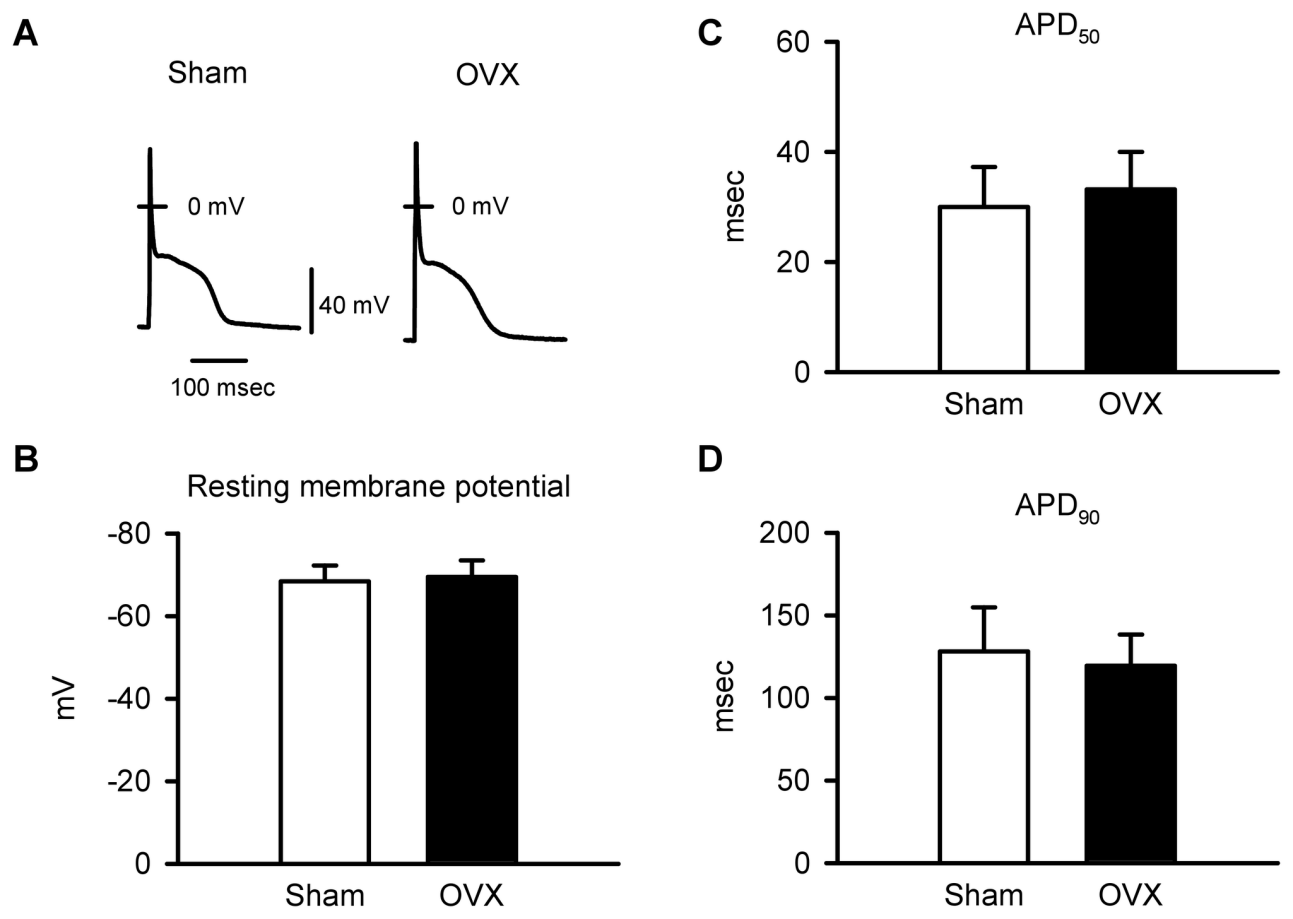
Action potential configurations were similar in myocytes from sham and OVX mice. **A**. Representative recordings of action potentials from sham and OVX myocytes. **B**. Mean values for resting membrane potentials were similar in myocytes from sham and OVX animals. **C**,**D**. APD_50_ and APD_90_ were not affected by long-term OVX (n=18 sham and 19 OVX myocytes; *p<0.05; t-test).

To establish the cellular basis for the increase in peak Ca^2+^ transients in OVX myocytes, specific EC-coupling mechanisms were evaluated. First, action potential configurations were compared in myocytes from sham and OVX mice during steady state pacing at 2 Hz. Figure 4A shows representative action potentials recorded from sham and OVX myocytes. Mean data indicate that the resting membrane potential (RMP) was similar in sham and OVX cells (Figure 4B). Action potential durations at 50 and 90% repolarization (APD_50_, APD_90_) also were similar in the two groups (Figure 4C,D). These data demonstrate that changes in cardiomyocyte action potential configuration were not responsible for the increase in Ca^2+^ release in OVX myocytes.

**Figure 5:**
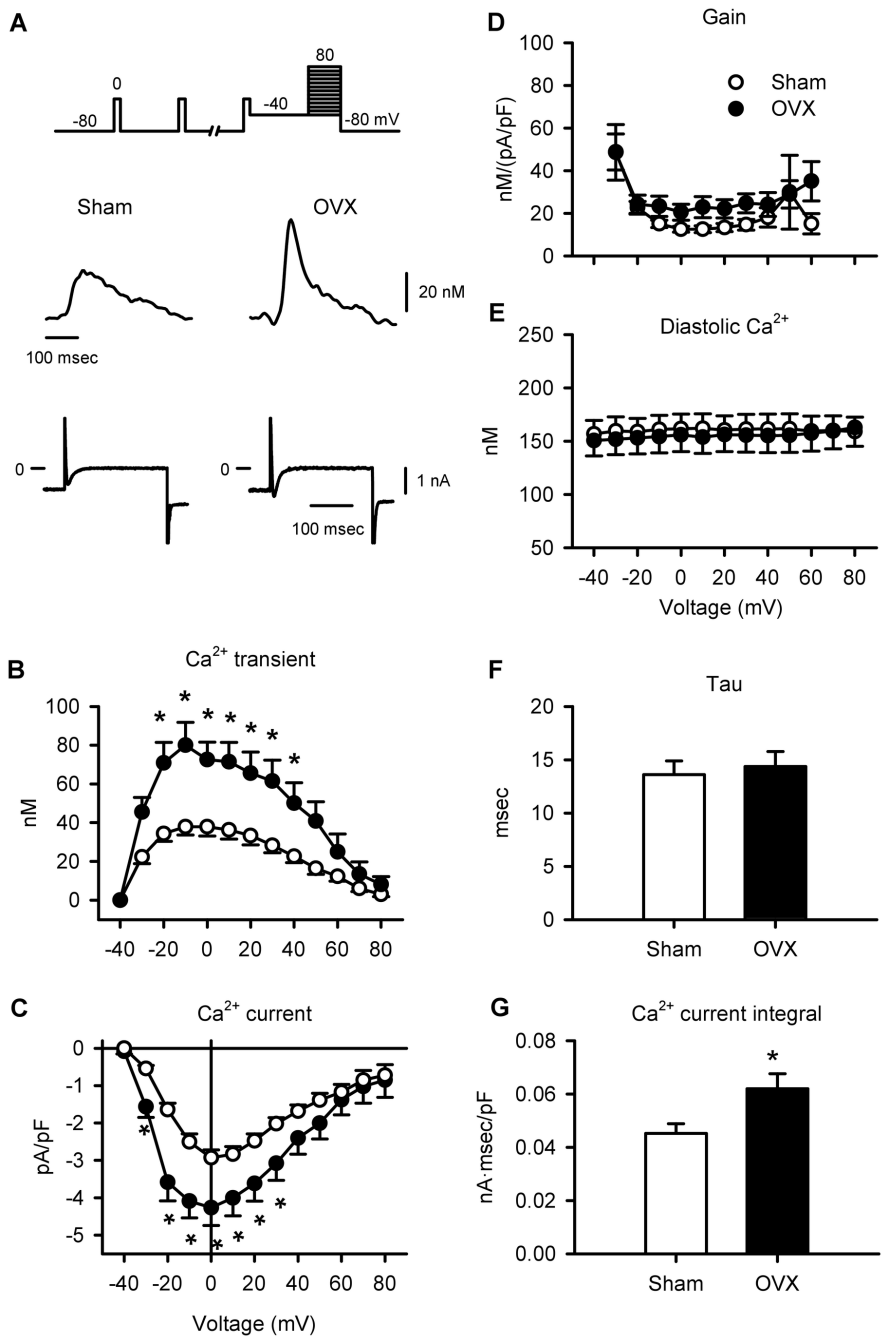
Long-term OVX increased Ca^2+^ currents and Ca^2+^ transients, but had no effect on EC-coupling gain. **A**. Voltage clamp protocol shown at top. Representative Ca^2+^ transients and Ca^2+^ currents recorded in myocytes from sham and OVX mice. **B**,**C**. Mean Ca^2+^ current densities and Ca^2+^ transients were increased by long-term OVX. **D**. The gain of SR Ca^2+^ release was similar in both groups. **E**. Diastolic Ca^2+^ levels were similar in the two groups at all voltages tested. **F**. The time course of inactivation (tau) for Ca^2+^ currents activated by a test step to 0 mV was similar in sham and OVX cells. **G**. The normalized integral of the Ca^2+^ current activated by a test step to 0 mV was larger in OVX myocytes compared to sham controls (n=14 sham and 16 OVX myocytes; *p<0.05)..

As SR Ca^2+^ release is proportional to the magnitude of the Ca^2+^ current, an increase in Ca^2+^ current could explain the larger Ca^2+^ transients in myocytes from aged OVX mice. To test this idea, voltage clamp experiments were conducted. Figure 5A shows representative Ca^2+^ transients and Ca^2+^ currents recorded from sham and OVX myocytes during a test step from -40 to 0 mV. Mean data show that OVX caused a marked increase in peak Ca^2+^ transients and Ca^2+^ currents (Figure 5B,C). This effect was dramatic, as OVX caused a 48% increase in Ca^2+^ current and a 91% increase in Ca^2+^ transients at the peak of the IV curve. However, because OVX increased both Ca^2+^ current and Ca^2+^ transients, the gain of EC-coupling (Ca^2+^ transient amplitude/Ca^2+^ current) was not affected by OVX (Figure 5D). By contrast, diastolic Ca^2+^ levels recorded in sham and OVX myocytes were similar at all test potentials examined (Figure 5E). To further evaluate the overall change in Ca^2+^ influx in sham and OVX myocytes, both the time constant for inactivation and the integral of the Ca^2+^ current (step to 0 mV) were compared in the two groups. The time constants were similar in sham and OVX myocytes (Figure 5F), but the integral of the Ca^2+^ current was larger in OVX cells when compared to sham-operated controls (Figure 5G). These observations indicate that larger Ca^2+^ currents in myocytes from aged OVX mice triggered a larger release of SR Ca^2+^ when compared to age-matched sham controls.

### Impact of long-term OVX on Ca^2+^ handling proteins

**Figure 6:**
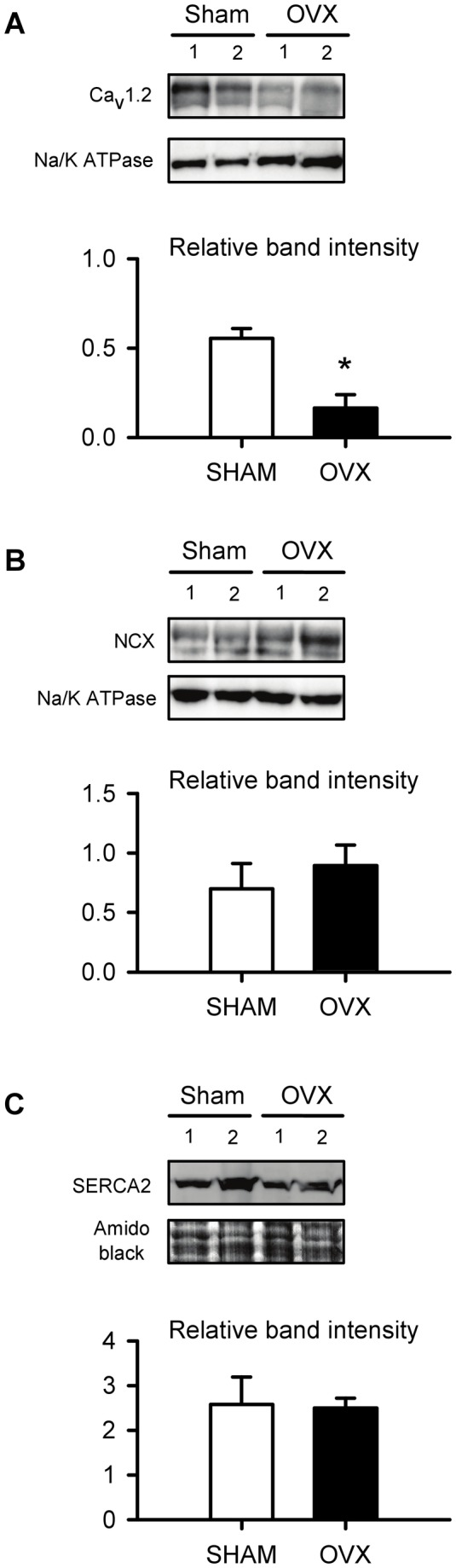
Expression of Ca^2+^ handling proteins in hearts from sham and OVX mice. **A**. Detection of ~240 kDa bands corresponding to Ca_V_1.2 in ventricular tissue from sham and OVX mice. Na/K ATPase (110 kDa band) was used as a loading control (15 µg of protein were loaded in each lane). Average Ca_V_1.2 band intensity was lower in OVX ventricular tissue compared to sham controls (n=3 hearts/group). **B**. Representative immunoblot illustrating ~116 kDa bands corresponding to NCX in sham and OVX ventricle. The loading control was Na-K ATPase as in A. Mean normalized NCX band intensity was similar in ventricular tissue from sham and OVX mice (n=3 hearts in each group). **C**. Detection of ~110 kDa bands corresponding to SERCA2 in the ventricles of sham and OVX mice. Amido black was used as a loading control (60 µg of protein were loaded in each lane). Average intensity of the SERCA2 bands was similar in sham and OVX ventricles (n=3 hearts in each group). In experiments where Na-K ATPase was used as a loading control, there was no significant difference in Na-K ATPase protein levels between sham and OVX (t-test, p=0.164) (*denotes p<0.05; t-test)..

To determine whether a change in the expression of Ca^2+^ handling proteins contributes to disruptions in Ca^2+^ homoeostasis in the aging OVX heart, we compared the expression of Ca_v_1.2, NCX and SERCA2 in sham and OVX hearts. As shown in Figure 6A, Ca_v_1.2 protein expression was actually reduced in the OVX group when compared to sham-operated controls. This indicates that an increase in the expression of Ca_v_1.2 does not account for the increased Ca^2+^ current in cardiomyocytes from OVX mice. We also compared the expression of NCX (Figure 6B) and SERCA2 (Figure 6C) in ventricles from sham and OVX mice. Results showed that the expression of NCX and SERCA2 was not affected by long term OVX in aging mice and suggest that increased expression of these proteins does not contribute to the enhanced rate of relaxation seen in cardiomyocytes from aged OVX animals.

### Long term OVX increases alters unitary SR Ca^2+^ release

**Figure 7:**
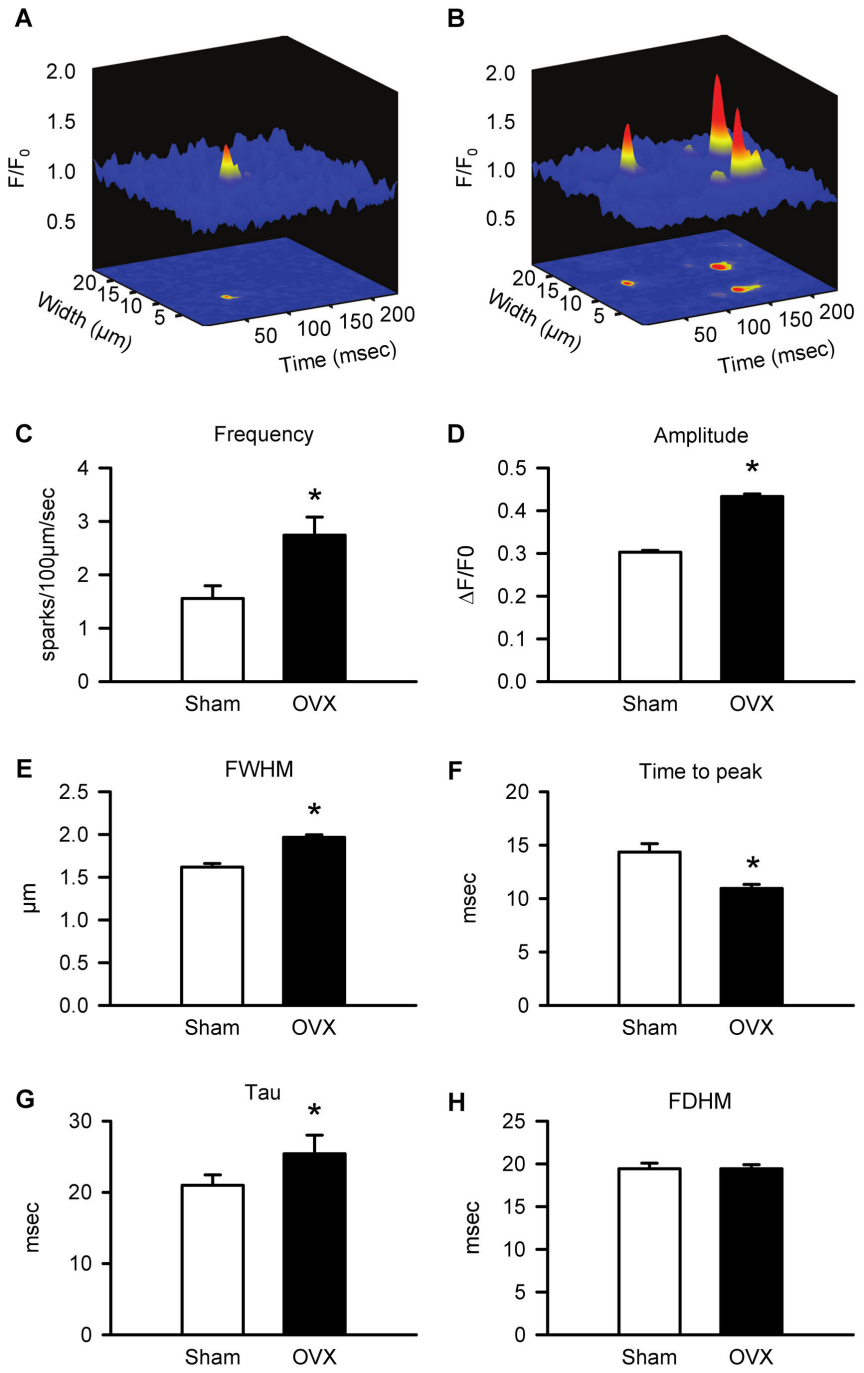
Ca^2+^ spark amplitudes, widths, and frequency were increased in OVX myocytes compared to sham controls. **A**,**B**. Representative Ca^2+^ sparks recorded in myocytes from sham and OVX animals. **C**,**D**,**E**. Long-term OVX increased mean spark frequency, amplitude, and full-width at half maximum (FWHM). **D**. Mean time-to-peak was reduced by OVX. **E**. Mean time constant of spark decay (tau) was prolonged by OVX. **F**. Full-duration at half maximum (FDHM) was similar in the two groups (n= 323 sham and n=780 OVX sparks; n=56 sham and n=74 OVX myocytes; *p<0.05; t-test).

To determine whether an increase in the size of unitary SR Ca^2+^ release events contributed to the increase in peak Ca^2+^ transients in cardiomyocytes from aged OVX mice, spontaneous Ca^2+^ sparks were compared in the two groups. Representative Ca^2+^ sparks recorded from sham and OVX myocytes are shown in Figure 7A and B. Mean data demonstrate that Ca^2+^ spark frequency was 76% higher in OVX cardiomyocytes when compared to sham controls (Figure 7C). Furthermore, Ca^2+^ sparks recorded in cells from aged OVX mice were 43% larger than those from sham controls (Figure 7D). Long-term OVX caused a 22% increase in spark width, measured as the full width half maximum (FWHM), when compared to sham myocytes (Figure 7E). Long-term OVX also affected the time course of Ca^2+^ sparks. While spark time-to-peak was reduced in myocytes from OVX mice (Figure 7F), the time constant of spark decay (tau) was prolonged (Figure 7G). However, the full duration at half maximum (FDHM) did not differ between the two groups (Figure 7H). Taken together, these data demonstrate that long-term OVX increases the amplitude and width of the unitary Ca^2+^ release events that underlie the Ca^2+^ transient. OVX also caused a marked increase in the frequency of Ca^2+^ sparks. The mechanism underlying this increase in spark frequency following long-term OVX was explored.

### Long-term OVX augments SR Ca^2+^ loading and promotes spontaneous SR Ca^2+^ release

**Figure 8:**
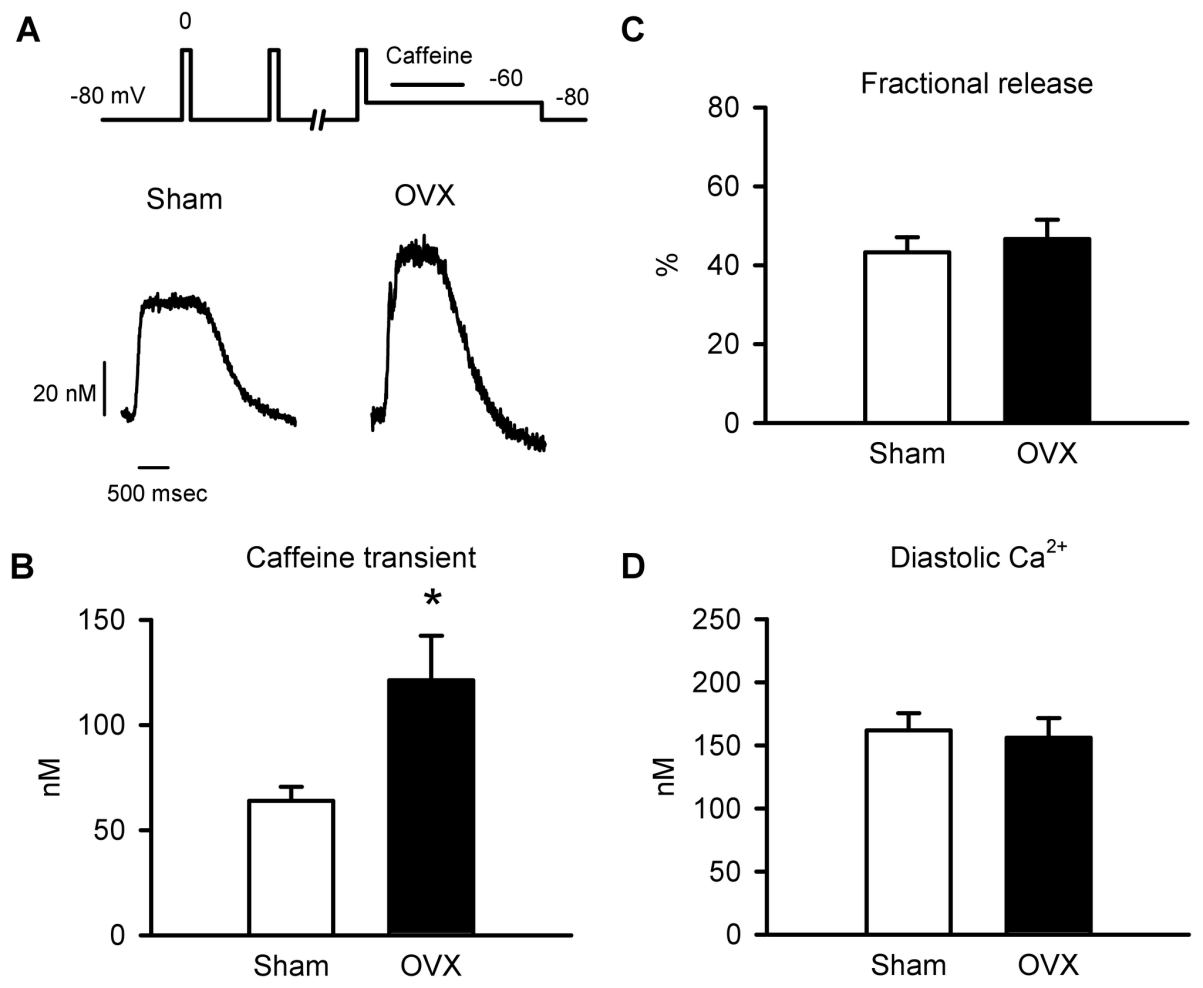
Long-term OVX increased SR Ca^2+^ stores. **A**. Representative Caffeine-induced Ca^2+^ transients in myocytes from sham and OVX mice. **B**. Caffeine-induced Ca^2+^ transients were larger in OVX myocytes than sham controls. **C**,**D**. Fractional SR Ca^2+^ release and diastolic Ca^2+^ levels were similar in the two groups (n=9 sham and 11 OVX myocytes; *p<0.05; t-test).

To determine whether the increase in spark frequency in cardiomyocytes from aged OVX mice was caused by an increase in SR Ca^2+^ content, intracellular Ca^2+^ stores were compared in myocytes from sham and OVX animals. Figure 8A shows representative caffeine-induced Ca^2+^ transients recorded from sham and OVX myocytes. The profile of these responses is comparable to caffeine-induced transients recorded under similar conditions in our previous studies [10,11,14,15]. Mean data showed that caffeine-induced Ca^2+^ transients were 90% larger in OVX myocytes compared to sham controls (Figure 8B). Long-term OVX increased both Ca^2+^ transients and caffeine-induced transients by approximately 90%, so OVX had no effect on fractional SR Ca^2+^ release (Ca^2+^ transient/caffeine-induced transient; Figure 8C). Furthermore, diastolic Ca^2+^ levels measured at -80 mV in these experiments were similar in myocytes from aged sham and OVX mice (Figure 8D). These data demonstrate that long-term OVX promoted SR Ca^2+^ loading, which can explain the increased Ca^2+^ spark frequency recorded in myocytes from OVX mice.

**Figure 9:**
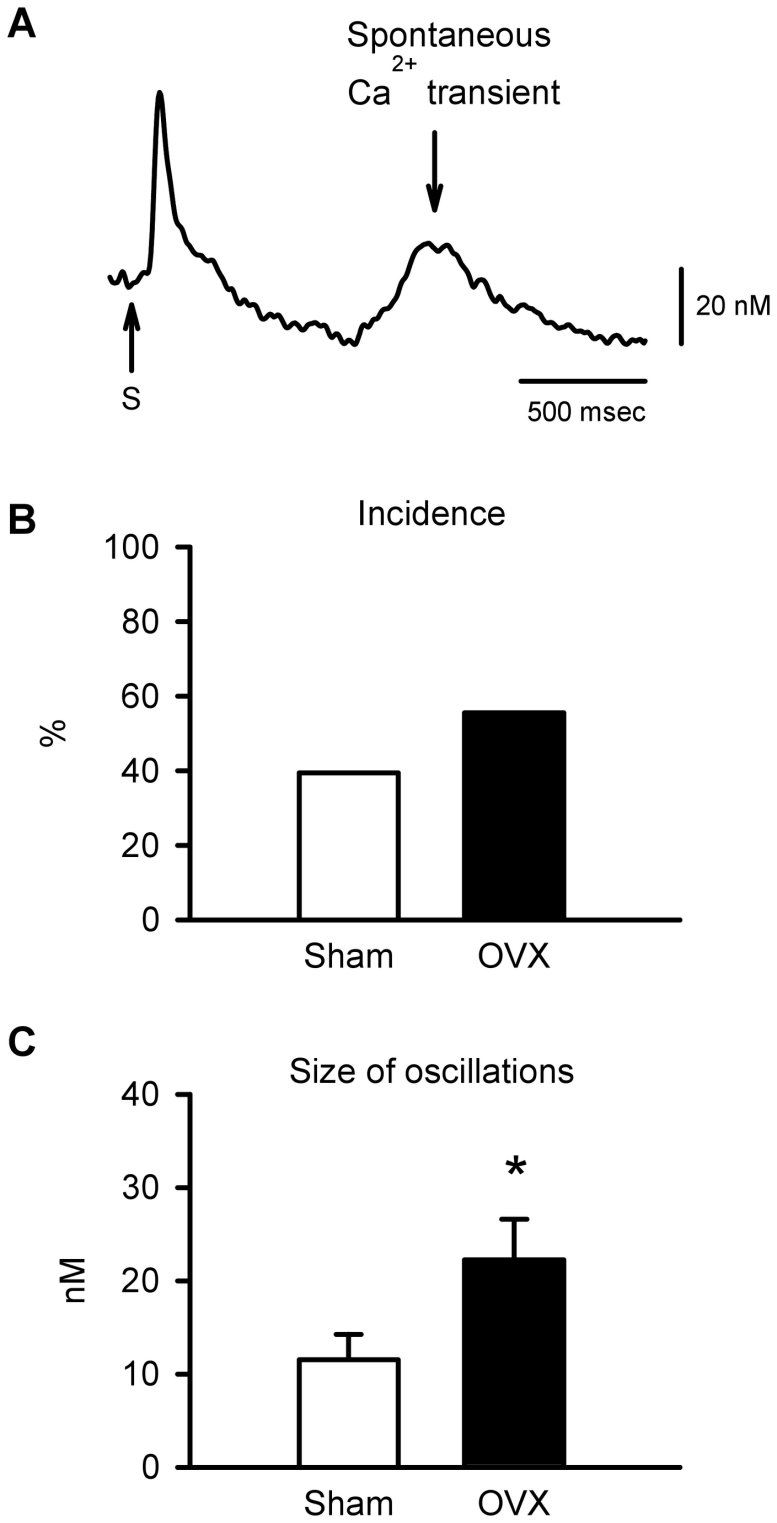
Long-term OVX increased spontaneous SR Ca^2+^ release. **A**. Representative recording of a spontaneous Ca^2+^ release followed a stimulated Ca^2+^ transient (S) in an OVX myocyte. **B**. The incidence of spontaneous Ca^2+^ transients was similar in myocytes from aged sham and OVX mice. **C**. Spontaneous Ca^2+^ transients were larger in OVX myocytes compared to sham controls (n=38 sham and 37 OVX myocytes; *p<0.05; t-test).

To determine whether elevated SR Ca^2+^ content in OVX myocytes enhanced spontaneous SR Ca^2+^ release, spontaneous Ca^2+^ transients were compared in myocytes from sham and OVX mice. Figure 9A shows a representative spontaneous Ca^2+^ transient following a stimulated transient (S) in an OVX myocyte. Long-term OVX did not significantly increase the incidence of spontaneous Ca^2+^ release when compared to sham controls (Figure 9B). However, OVX significantly increased the magnitude of spontaneous Ca^2+^ transients by 93% when compared to sham controls (Figure 9C). These data confirm that long-term OVX increased SR Ca^2+^ loading and promoted spontaneous SR Ca^2+^ release in the aging heart.

## Discussion

Our study provides the first evidence that cardiomyocyte Ca^2+^ homeostasis and contractile function are altered by long-term OVX in aging female mice. Myocytes from aging OVX mice had larger and faster Ca^2+^ transients when compared to sham operated controls. Interestingly, this dramatic increase in SR Ca^2+^ release did not enhance contractile function either in ventricular myocytes or *in vivo*. Thus, long-term OVX disrupted the relationship between intracellular Ca^2+^ and cardiac contraction in the aging heart. We examined the underlying mechanisms involved and showed that long-term OVX reduced myofilament Ca^2+^ sensitivity in the aging heart. Results also demonstrated that increased Ca^2+^ current density along with larger, wider Ca^2+^ sparks amplified Ca^2+^ transients in myocytes from OVX mice. The increased Ca^2+^ current in OVX myocytes was not due to an increase in Ca^2+^ channel protein expression. Furthermore, the rapid decay of the Ca^2+^ transient in OVX cells was not due to increased expression of NCX or SERCA2. However, elevated intracellular Ca^2+^ in OVX cells led to higher SR Ca^2+^ loads, increased spark frequency and spontaneous SR Ca^2+^ release. Thus, myocytes subjected to long-term OVX exhibit reduced myofilament Ca^2+^ responsiveness, Ca^2+^ dysregulation and spontaneous SR Ca^2+^ release.

Previous studies have shown that SR Ca^2+^ release and contractions are larger in cardiomyocytes from young adult males when compared to age-matched females (reviewed by [34]). Interestingly, short-term removal of ovarian estrogen increases Ca^2+^ transients in myocytes from young adult females [15-19] but cf. [20,21], which suggests that short-term OVX leads to changes in SR Ca^2+^ release consistent with conversion to a male phenotype. As peak contractions and Ca^2+^ transients decline with age in cardiomyocytes from males but not females [10,11], it is possible that long term OVX actually suppresses SR Ca^2+^ release in the aging female heart. However, the present study reports the novel finding that long-term OVX did not suppress SR Ca^2+^ release but rather enhanced it, even in very old female mice. Thus, long-term deficiency of ovarian-derived estrogen, including the lack of normal pubertal estrogen, does not produce changes in SR Ca^2+^ release consistent with conversion of a female to a male phenotype. The impact of age on contraction in older males might be due to lower testosterone levels [35] that can inhibit SR Ca^2+^ release and suppress contractions, as seen in myocytes from younger males after gonadectomy [36,37].

We and others have previously shown that SR Ca^2+^ release is augmented in cardiomyocytes from young adult mice, 3-26 weeks after OVX [34]. In young OVX mice, we found that increased SR Ca^2+^ release was not due to an increase in Ca^2+^ current. Instead, this was the result of higher gain due to elevated SR Ca^2+^ content and an increase in the amplitude of Ca^2+^ sparks [15]. Table 3 summarizes the major effects of OVX on key Ca^2+^ handling parameters in young adult OVX mice as reported in our previous study [15]. The present study extends these findings to demonstrate that enhanced SR Ca^2+^ release persists in the aging heart, even almost two years after OVX. A key observation in this study is that effect of OVX on specific mechanisms involved in cardiomyocyte homoeostasis differed markedly in the aging heart when compared to young adult hearts, as shown in Table 3 that compares our present study and our earlier work [15]. While the impact of OVX on SR Ca^2+^ content was similar regardless of age, Ca^2+^ transients increased in parallel with Ca^2+^ current in aged OVX myocytes, with no effect on the gain of SR Ca^2+^ release. As the magnitude of SR Ca^2+^ release is proportional to the size of the Ca^2+^ current [38], our results indicate that, unlike young OVX mice, an increase in Ca^2+^ current is a key mechanism responsible for augmenting SR Ca^2+^ release following long-term OVX in the aging heart. We also demonstrated that the increase in peak Ca^2+^ current was not due to an increase in the expression of Ca_v_1.2, which has been reported in hearts from younger animals subjected to short term OVX [39]. Indeed, we showed that Ca_v_1.2 expression was actually reduced following long-term OVX in the aging heart. The increase in the activity and expression of protein kinase A (PKA) that occurs following OVX [40] may be implicated in the increase in peak Ca^2+^ current, as discussed in more detail below.

**Table 3:** Comparison of Key Ca^2+^ Handling Mechanisms in Hearts and Cardiomyocytes From Young Adult OVX and Aged OVX Female Mice.

**Parameter**	***YoungOVX***	***YoungOVX***	***AgedOVX***	***AgedOVX***
	***Relativetosham***	***% change***	***Relativetosham***	***% change***
Ca^2+^ transient, peak	⬆	106	⬆	91
Ca^2+^ transient, rate of rise	⬆	54	⬆	43
Ca^2+^ transient, rate of decay	⬆	113	⬆	71
Ca^2+^ current	⬌	⬌	⬆	48
Gain of SR Ca^2+^ release	⬆	112	⬌	⬌
SR Ca^2+^ content	⬆	83	⬆	90
Diastolic Ca^2+^	⬌	⬌	⬌	⬌
Ca^2+^ spark frequency	⬆	25	⬆	76
Ca^2+^ spark amplitude	⬆	11	⬆	43
Ca^2+^ spark width	⬌	⬌	⬆	22
Ca^2+^ spark duration	⬌	⬌	⬌	⬌
RMP			**⬌**	⬌
APD_50_, APD_90_			**⬌**	⬌
Myocyte contraction, peak			**⬌**	⬌
Myocyte contraction, time to peak			⬇	18
Myocyte contraction, time to 50% relaxation			⬇	25
Fractional shortening, *in vivo*			**⬌**	⬌
Ejection fraction, *in vivo*			**⬌**	⬌
Heart rate, *in vivo*			**⬌**	⬌
Maximum actomyosin ATPase activity			**⬌**	⬌
Myofilament Ca^2+^ sensitivity (EC_50_)			⬇	26
Ca_v_1.2 expression			⬇	70
NCX expression			**⬌**	**⬌**
SERCA2 expression			**⬌**	**⬌**

Young adult data are from reference [15]. ⬆ or ⬇ represents increase or decrease relative to age-matched sham control. ⬌ represents no change relative to age-matched sham control. The magnitude of change for each parameter relative to age-matched sham controls is indicated in the column entitled "% change".

The results of the present study also demonstrate that that long-term OVX had much more dramatic effects on Ca^2+^ spark properties than short-term OVX, as shown in Table 3. While OVX in young adult mice increased spark amplitude by 11% [15], long-term OVX increased spark amplitude by 43% and spark width by 22%, resulting in a marked increase in the overall size of these release events. Long-term OVX also modified the spark time course, by abbreviating time-to-peak and prolonging spark decay, although these changes did not affect overall spark duration. However, the shorter time-to-peak may underlie the increased rate of rise of the Ca^2+^ transient and shorter time-to-peak contraction observed in OVX myocytes in this study. Together, these observations suggest that a marked increase in the size of individual Ca^2+^ release events contributes to enhanced SR Ca^2+^ release following long-term ovarian estrogen deprivation in the aging heart.

As OVX increases SR Ca^2+^ release, it would be expected to increase the magnitude of cardiac contraction, although this was not explored in our earlier study (Table 3 [15]). A novel and important finding in the present study was our observation that the relationship between intracellular Ca^2+^ and contraction was dramatically altered by long-term OVX in the aging mouse heart. Even though peak Ca^2+^ transients were increased by 91% following long-term OVX, this did not augment cardiomyocyte contraction, even when cells were paced at rapid stimulation frequencies. Furthermore, this marked increase in SR Ca^2+^ release at the myocyte level had no effect on cardiac contractile function *in vivo*, as measured by echocardiography. We found that the underlying mechanism was a decrease in myofilament Ca^2+^ sensitivity in the aging heart. Reduced myofilament Ca^2+^ sensitivity would normally be expected to reduce cardiac contractile function. However, contractile function was preserved by a marked increase in the amount of Ca^2+^ available to the myofilaments in the aged, estrogen-deprived heart. Our observation that contractile function is maintained by elevated Ca^2+^ in the aging, estrogen-deprived female heart may help explain why older women are predisposed to heart failure with preserved ejection fraction rather than systolic heart failure, which occurs characteristically in older men [41].

Elevated SR Ca^2+^ content in the aging OVX heart could arise from increased Ca^2+^ influx, as reported here, together with increased SERCA2a levels. The rapid decay rates of Ca^2+^ transients and contractions observed here and previously [16,17] are compatible with increased SR Ca^2+^ uptake and/or extrusion in OVX myocytes. However, as shown in the present study and previously, the abundance of SERCA2 is unaffected by OVX [17,39,42] and some studies have reported it is actually reduced by OVX [43-45]. Furthermore, as demonstrated in our study and previously, the expression of NCX is either unaffected by OVX [17,43] or is reduced [39,42]. Together these observations indicate that the rapid decay of the Ca^2+^ transients observed following long-term OVX is not due to an increase in the expression of SERCA2 or NCX. However, it is possible that the observed decline in myofilament Ca^2+^ sensitivity explains the rapid decay of Ca^2+^ transients in OVX myocytes. A decrease in myofilament Ca^2+^ sensitivity could make intracellular Ca^2+^ more rapidly available to SERCA in the OVX heart.

When SR Ca^2+^ load is high, the SR releases Ca^2+^ in the form of spontaneous Ca^2+^ sparks to limit SR Ca^2+^ content [46]. It is likely that the increase in Ca^2+^ spark frequency we observed in myocytes from aged OVX mice occurred in response to the marked increase in SR Ca^2+^ load. Previous studies have shown that advanced age increases SR Ca^2+^ content in myocytes from female rodents [10,11]. The results of the present study suggest that increased SR Ca^2+^ load in aged female myocytes is mediated by declining estrogen levels in aged female rodents [47]. It is well established that high levels of SR Ca^2+^ lead to Ca^2+^ overload and induce spontaneous Ca^2+^ release from the SR [48]. Indeed, we observed spontaneous SR Ca^2+^ release in myocytes from both sham and OVX animals, although the magnitude of this effect was significantly larger in the OVX group. Ca^2+^ overload and spontaneous SR Ca^2+^ release can disrupt myocardial function and lead to abnormal electrical and contractile activity [48,49]. Our results demonstrate that ovarian estrogen deprivation leads to profound Ca^2+^ dysregulation and the initiation of spontaneous SR Ca^2+^ release. It is possible that disruptions in myocardial Ca^2+^ homeostasis induced by long-term estrogen deprivation may increase susceptibility to cardiovascular diseases such as arrhythmias in the aging female heart.

Estrogen levels have been shown to decline with age in female rodent models [47] and this is exacerbated in the setting of OVX [50,51]. This suggests that the profound Ca^2+^ dysregulation observed in the aging OVX heart is linked to low estrogen levels. Still, the pathway by which estrogen may modify Ca^2+^ handling has not yet been identified. We found no evidence for increased expression of the key Ca^2+^ handling proteins Ca_v_1.2, NCX or SERCA2, which suggests that post-translational modifications of these and possibly other proteins may be important. One central pathway in the regulation of Ca^2+^ handling in cardiomyocytes is the cAMP/ PKA pathway. Previous studies have shown an increase in both basal and β-agonist stimulated-PKA activity in hearts from rats 6 weeks after bilateral OVX [40] as well as an increase in PKA expression [17]. Furthermore, estrogen replacement has been shown to restore PKA activity and expression levels to control values [17,40]. Increased PKA activity is known to phosphorylate various downstream targets [38] and could modify Ca^2+^ handling in the OVX heart in a manner consistent with that seen in the present study. For example, L-type Ca^2+^ channel phosphorylation will increase peak Ca^2+^ current and thereby increase Ca^2+^-induced Ca^2+^ release from the SR [52]. Furthermore, phosphorylation of phospholamban will increase SERCA2a activity and thus enhance the rate of SR Ca^2+^ uptake [53]. Phosphorylation of troponin I at N-terminal serines promotes faster relaxation by facilitating dissociation of Ca^2+^ from the myofilaments and reducing myofilament Ca^2+^ sensitivity [54]. Phosphorylation of the ryanodine receptor, although controversial [55], could help explain the increase in Ca^2+^ spark amplitudes reported in the present study. Increased PKA activity may be particularly important *in vivo*, as there is evidence that OVX enhances depolarization-induced norepinephrine release and elevates sympathetic tone in the heart [56,57]. Stimulation of the cAMP/PKA pathway also could account for the increase in spontaneous Ca^2+^ release in myocytes from aged OVX mice and could promote arrhythmias in the aging female heart. Further exploration of the role of this cAMP/PKA pathway in modifying Ca^2+^ handling in the OVX heart could be illuminating.

There is recent evidence that the production of reactive oxygen species (ROS) is increased in the aged OVX heart [51] and this may explain some of our findings. For example, increased ROS activity has been shown to increase ryanodine receptor activity, which could contribute to the increase in Ca^2+^ sparks reported in our study [58]. Increased ROS activity also has been reported to increase peak Ca^2+^ current in some models [58] and could contribute to the enhanced Ca^2+^ current we observed in myocytes from aged OVX mice. Furthermore, as oxidative stress has been shown to reduce myofilament Ca^2+^ sensitivity in the heart [59], it is possible that an increase in production of ROS leads to the decrease in myofilament Ca^2+^ sensitivity observed in our study. On the other hand, previous studies have reported that short-term (10 weeks) OVX increases myofilament Ca^2+^ sensitivity [60], so prolonged ovarian estrogen withdrawal may be required to desensitize myofilaments in the aging female heart. ROS also reduces SERCA2 activity, which is not compatible with the faster time courses of contraction and Ca^2+^ transients observed in our study [58,61]. Additional experiments to investigate the role of ROS in Ca^2+^ dysregulation in the estrogen-deprived heart could be informative.

Previous studies have provided evidence that OVX can modify the structure of the heart. While heart weight-to-body weight ratios are similar [42,43,51,62,63], echocardiography has revealed increased IVSd thickness, increased wall thickness and reduced LVID in young adult mice after 10 weeks of OVX [42]. In agreement with previous studies in mice and rats [42,43,51,62,63], we found that ventricle weight-to-body weight ratios were similar in sham and OVX mice. By contrast, long-term OVX had no effect on LVPW thickness or LVID measured in systole or diastole and actually reduced IVSd thickness. These observations suggest the structural changes in the whole heart observed early after OVX may be transient. The effects of OVX cardiomyocyte structure may also depend on the duration of ovarian steroid withdrawal. Shorter periods of steroid withdrawal (e.g. <26 weeks) have no effect on cardiomyocyte capacitance [15]. However, we found that long term OVX reduced cell capacitance and cell volume, thereby reducing cardiomyocyte membrane area. Whether this is due to long term remodeling of membranes such as the t-tubules or caveolae remains to be determined.

There are limitations to the experimental approaches used in this study. Our studies did not investigate OVX mice treated with estrogen replacement, although this would be an interesting area for additional investigation in future studies. In addition, we removed the ovaries early in life to determine whether long-term estrogen withdrawal would result in an aging phenotype characteristic of that seen in aging males with respect to Ca^2+^ handling [9-11]. In consequence, the period of estrogen withdrawal was prolonged and the effects on myocardial Ca^2+^ homeostasis were dramatic. This also produced a model that was characterized by the lack of exposure of the heart to normal pubertal systemic estrogen modeling. This model of early ovarian steroid withdrawal has been used previously in other studies [12,15,63], but it contrasts with the more commonly used approach where the effects of OVX are investigated in adult animals where tissues have already been exposed to estrogen. Additional experiments with other time frames for estrogen deprivation could be explored in the future. Although the ovaries are the primary source of estrogens, other tissues such as adipose tissue, vascular tissue and bone, express the enzyme aromatase that can convert testosterone to 17ß-estradiol [64]. These non-gonadal sources of estrogen could be important in OVX animals. Furthermore, aromatase is expressed in neonatal tissues and cardiomyocytes [65,66] and in the adult rodent heart [67]. It is possible that androgens can be locally converted to estrogens in the myocardium [67]. Whether exposure of cardiomyocytes to sex steroid hormones can be regulated at the local tissue level is an important area for further investigation.

In summary, our study showed that long-term deprivation of ovarian estrogen disrupted myocyte Ca^2+^ homeostasis and contractile function in the aging female heart. Although Ca^2+^ transients were larger in OVX myocytes, *in vitro* and *in vivo* fractional shortening were similar in sham and OVX mice. The underlying mechanism involved a decrease in myofilament Ca^2+^ sensitivity in the aging OVX heart. The increase in peak Ca^2+^ transients in OVX myocytes was mediated by an increase in both Ca^2+^ current and the size of unitary Ca^2+^ release events. Higher intracellular Ca^2+^ led to an increase in SR Ca^2+^ load, an increase in spark frequency and spontaneous SR Ca^2+^ release. These results demonstrate that long-term ovarian estrogen deprivation reduces myofilament Ca^2+^ sensitivity, promotes Ca^2+^ dysregulation, and increases spontaneous Ca^2+^ release in the aging female heart.

## Supporting Information

Methods S1 **Supplemental methods.**
(DOCX)
