# Supplementary material for: The Impact of Ovariectomy on Calcium Homeostasis and Myofilament Calcium Sensitivity in the Aging Mouse Heart
Source: PLoS One. 2013 Sep 18;8(9):e74719. doi: 10.1371/journal.pone.0074719 (PMC3776741; doi:10.1371/journal.pone.0074719)
Supplement: Methods S1 — Supplemental methods. (DOCX) [file pone.0074719.s001.docx]

**SUPPLEMENTAL METHODS**

**Animals.**  All animal protocols conformed to Canadian Council on Animal Care guidelines (Ottawa, ON: Vol. 1, 2^nd^ edition, 1993; Vol. 2, 1984) and were approved by the Dalhousie University Committee on Laboratory Animals. Female C57BL/6 mice were obtained from Charles River Laboratories (St. Constant, QC) following an OVX or sham surgery at one month of age. Animals were housed in micro-isolator cages at Dalhousie University and maintained on a 12-hour light/dark cycle, with free access to food and water, until use at 20-24 mos.

**Myocyte Isolation.** Ventricular myocytes were isolated by enzymatic dissociation as described previously [[1](#_ENREF_1)]. Animals were weighed then anaesthetized with sodium pentobarbital (IP, 220 mg/kg) plus heparin (IP, 3000 U/kg); the adequacy of anesthesia was confirmed by the absence of pedal withdrawal and corneal reflexes. Hearts were perfused through the aorta (2 ml/min, 10 min) with buffer (mM): 105 NaCl, 5 KCl, 25 HEPES, 20 glucose, 0.33 NaH_2_PO_4_, 1.0 MgCl_2_, 3.0 Na-pyruvate, 1.0 lactic acid (pH 7.4, 100% O_2_, 37°C). Hearts were then perfused with the same buffer plus protease dispase II (Roche Diagnostics, Laval, QC; 0.10-0.15 mg/ml), collagenase type 1 (250 U/mg, Worthington, Lakewood, NJ; 0.3-0.5 mg/ml) and trypsin (Sigma Aldrich, Oakville, Ont; 0.01-0.02 mg/ml) for 8-10 min. The ventricles were isolated and minced in high potassium buffer (mM): 80 KOH, 30 KCl, 3 MgSO_4_.7H_2_O, 30 KH_2_PO_4_, 50 L-glutamic acid, 20 taurine, 0.5 EGTA, 10 HEPES, 10 glucose (pH 7.4 with KOH). The suspension was filtered through a 225 µm polyethylene filter. Experiments used quiescent, rod shaped, striated myocytes with no visible membrane damage. The uterus was removed from each animal, dried and weighed to confirm the success of OVX.

**Functional assessment of cardiomyocytes.** Contractions, transmembrane currents and Ca^2+^ transients were measured simultaneously as described in our previous studies [[2](#_ENREF_2)]. Myocytes were loaded with fura-2 AM (5 μM) and incubated in the dark for 20 min (20^o^C). Cells were placed in a chamber on an inverted microscope and superfused (3 ml/min) with the following buffer (mM): 135 NaCl; 10 glucose; 10 HEPES; 4 KCl; 1 CaCl_2_; 1 MgCl_2_ (pH 7.4 with NaOH). In voltage clamp experiments, the buffer solution contained 4‑aminopyridine (4 mM) to inhibit transient outward K^+^ current. Test steps were made from -40 mV to inactivate Na^+^ current. As hypothermia (e.g. 22^o^C) causes a profound increase in contractions, Ca^2+^ transients, SR Ca^2+^ stores and EC-coupling gain [[3](#_ENREF_3)], all experiments were performed at physiological temperature (37°C).

Contractions were recorded as unloaded cell shortening at 120 Hz with a video edge detector (Crescent Electronics, Sandy, UT) coupled to a CCD camera (model TM-640, Pulnix America). Fura-2 was excited at 340 and 380 nm and fluorescence emission was measured at 510 nm with a DeltaRam fluorescence system (Photon Technology International (PTI), Birmingham, NJ) and Felix software (version 1.4, PTI). Fluorescence emission at each excitation wavelength was measured at a sampling interval of 5 msec. Emission ratios (340/380 nm) were converted to Ca^2+^ concentrations with an *in vitro* calibration curve as in our previous studies [[4](#_ENREF_4),[5](#_ENREF_5)]. When contractions and fluorescence were recorded simultaneously, a dichroic cube was used to direct red light to the camera/edge detector system and the remaining light to the photomultiplier tube.

In field stimulation experiments, cells were stimulated at 2 Hz with 3 msec pulses delivered through platinum electrodes. In some experiments, cells were also paced at 8 Hz. Pulses were generated by a stimulus isolation unit (SIU- 102; Warner Instruments, Hamden, CT) controlled with pClamp software (version 8.2; Molecular Devices, Sunnyvale, CA). Membrane potentials and transmembrane currents were recorded with microelectrodes (15-25 MΩ) filled with filtered 2.7 M KCl. Experiments used discontinuous single electrode voltage clamp (5-8 kHz) with an Axoclamp 2B amplifier (Molecular Devices, Sunnyvale, CA) and pClamp software. In all voltage clamp studies, we monitored the switching circuit to ensure adequate settling time for accurate voltage measurement. We previously confirmed that the membrane potential measured by a single electrode during switch clamp accurately measured the membrane potential with a second independent microelectrode [[6](#_ENREF_6)]. To generate current-voltage (IV) curves, cells were held at ‑80 mV and trains of five 50 msec conditioning pulses from -80 to 0 mV (2 Hz) preceded test steps. After the last conditioning pulse, cells were repolarized to ‑40 mV then depolarized to voltages between -40 and +80 mV to elicit the Ca^2+^ current. In some experiments, we confirmed the identity of the Ca^2+^ current. Currents were recorded in the absence and presence of a rapid switch to cadmium (200 µM), to block Ca^2+^ current. Cadmium blocked 92.5 ± 4.9% of the Ca^2+^ current (n=3) as in our previous studies [[2](#_ENREF_2),[5](#_ENREF_5)]. Action potentials were measured in separate experiments, in myocytes that were not loaded with fura-2. Cells were paced with trains of twenty 3 msec pulses at 2 Hz.

SR Ca^2+^ load was measured by rapid application of 10 mM caffeine (37^o^C). In these experiments, cells were voltage clamped as described above and repolarized to -60 mV after the conditioning pulses. After 500 msec, caffeine was applied for 1 sec in the following buffer solution (mM): 10 caffeine; 140 LiCl; 4 KCl; 10 glucose; 5 HEPES; 4 MgCl_2_; 4 4-aminopyridine; 0.3 lidocaine. Caffeine was prepared in Na^+^ and Ca^2+^-free solution to inhibit Ca^2+^ extrusion via Na^+^-Ca^2+^ exchange [[7](#_ENREF_7),[8](#_ENREF_8)]. Estimates of SR Ca^2+^ stores were the same when cells were briefly exposed to Ca^2+^- and Na^+^-free solution before the switch to caffeine, as we have shown previously [[3](#_ENREF_3)].

Ca^2+^ sparks were measured as described previously [[4](#_ENREF_4)]. Myocytes were loaded with Fluo-4 AM (20 μM, 30 min, 20^o^C; Invitrogen, Burlington, ON). Cells were placed in a laminin-coated chamber (1 mg laminin/100 ml M199 medium) on a laser scanning confocal microscope (Zeiss LSM 510-Meta, Carl Zeiss Canada Ltd, Toronto, ON). Myocytes were superfused with buffer (mM): 135.5 NaCl, 4 KCl, 10 HEPES, 1 MgCl_2_, 10 glucose, 1 CaCl_2_, 2 probenecid, (pH 7.4; 37°C; 4 mL/min). A 63x oil immersion lens (Plan-Apochromat DIC objective, NA 1.40) was used to perform experiments. Quiescent myocytes were scanned for 6 s. The argon laser (488 nm) and collection of line scan images (525 nm, pinhole size=98 μm, scan speed=649.35 lines/s, 512 pixels/line, laser intensity=20%) were controlled by LSM software (version 3.2, Carl Zeiss Canada Ltd). Line scan images were analyzed for spontaneous Ca^2+^ sparks with the SparkMaster plug-in [[9](#_ENREF_9)] for ImageJ software (v1.34, NIH). SparkMaster parameters were: scanning speed=649.35 lines/s; pixel size (μm)=cell length/512 pixels; background=10; criteria=3.8; number of intervals=1; output=F/F0 + sparks, extended kinetics (where F=fluorescence intensity, F0=background). Each image was inspected manually to exclude anomalies (*e.g.* extended bright lines, clusters of sparks) that were detected as sparks.

**Echocardiography.** In some experiments, two-dimensional guided M-mode echocardiography was performed. Mice were anesthetized with 2% isoflurane in oxygen, placed on a 37°C platform and electrocardiography (ECG) electrodes (Grass technologies, RI, USA) were placed subcutaneously. A high-resolution linear transducer (i13L, GE ultrasound, Horten, Norway) connected to a Vivid 7 imaging system (GE Medical Systems, Horten, Norway) was used to assess the mice. M-mode (short axis) was used to generate images and ECG traces were used to determine heart rate. Measurements of diastolic and systolic ventricular dimensions were measured offline to generate the following parameters: interventricular septum thickness in diastole (IVSd) and systole (IVSs), left ventricular internal diameter in diastole (LVIDd) and systole (LVIDs), left ventricular posterior wall thickness in diastole (LVPWd) and systole (LVPWs), fractional shortening and ejection fraction.

**Assessment of myofilaments.** Animals were weighed and anesthetized as described above. The ventricles were then removed, weighed to calculate ventricular weight-to-body weight ratios and quickly frozen at -80^o^C. Myofilaments were isolated as described [[10](#_ENREF_10)] and stored at -80°C until use. Then they were homogenized in ice cold standard buffer (mM, 60 KCl, 30 imidazole, 2 MgCl_2_, 0.01 leupeptin, 0.1 PMSF, 0.2 benzamidine, 0.1 cantharidin; pH 7.0) and centrifuged at 14,100 X g (15 min, 4°C). The pellets were resuspended for 45 min in ice cold standard buffer plus 1% Triton X-100, then centrifuged at 1,100 X g (15 min, 4^o^C) and the pellets were washed three times in ice cold standard buffer. Myofilaments were stored on ice for up to 2 h.

Actomyosin MgATPase assay was determined as described previously [[10](#_ENREF_10)]. Myofilaments (25 mg) were incubated in activating solutions containing varying levels of free Ca^2+^ as described earlier [[11](#_ENREF_11)]. Myofilaments were incubated in these solutions for 10 min (32°C), then reactions were quenched with 10% trichloroacetic acid. Inorganic phosphate production was measured by adding an equal volume of 0.5% FeSO_4_ and 0.5% ammonium molybdate in 0.5 M H_2_SO_4_, and reading the absorbance at 630 nm.

**Western blots.** The ventricles were isolated as described above. Ice cold protein extraction buffer (5 mM EDTA, 20 mM HEPES, 2% LDS, 10% glycerol, 1mM AEBSF, 800 nM apoprotin, 20 µM leupeptin, 10 µM pepstatin, 50 µM bestatin, 15 µM E-64) was added to the tissue. The tissue was homogenized for 60 s, flash frozen in liquid nitrogen and then sonicated 3 times with a microtip sonicator set to 30% max output. Samples were flash frozen after each sonication step. Next, the samples were heated (5 mins, 70^o^C) then cooled on ice (5 mins). Samples were then centrifuged at 12,000 rpm (8 mins, 4^o^C) and the supernatant was aliquoted, flash frozen and stored at -80^o^C. Protein concentration was determined with a DC Protein Assay (BioRad).

For the detection of Ca_v_1.2 protein, rabbit anti- Ca_v_1.2 polyclonal antibody (Alomone, ACC-003-AG) was used at a dilution of 1:2000. For NCX detection, mouse anti-NCX monoclonal antibody (SWANT, R3F1) was used at 1:1000. For the detection of SERCA2, mouse anti-SERCA2 monoclonal antibody (Affinity Bioreagents, MA3-919) was used at 1:2000. This antibody recognizes SERCA2a and b isoforms (but not SERCA 1 or SERCA 3) and has been extensively used to investigate the expression of SERCA2 in the heart (*e.g*. [[12](#_ENREF_12),[13](#_ENREF_13)]). For the detection of Na/K ATPase protein, rabbit anti-Na/KATPase polyclonal antibody (Abcam) was used at 1:2000. Secondary antibodies used in these experiments were goat anti-mouse horseradish peroxidase-conjugated polyclonal antibody (Abcam, 1:30,000) and goat anti-rabbit horseradish peroxidase-conjugated polyclonal antibody (Abcam, 1:30,000).

Protein was loaded and resolved by an 8% polyacrylamide gel. Proteins were transferred to nitrocellulose membrane and blocked with blocking buffer [1% bovine serum albumin in a Tris-Buffered Saline Tween-20 (TBST)] for 1 h at room temperature. For immunoblots, the membranes were incubated in blocking buffer overnight (4^o^C) with the primary antibody of interest. Anti-Na/K ATPase antibody was used as a loading control for Ca_v_1.2 and NCX experiments after stripping the blots, as in previous studies [[14](#_ENREF_14)]. For SERCA2 experiments, the total protein stain amido black was used as a loading control as in previous studies [[15](#_ENREF_15)]. Membranes were then washed in TBST and incubated with appropriate species-specific horseradish peroxidase-conjugated secondary antibodies (Abcam) for 2 h at room temperature. The membranes were washed with TBST (3 washes, 10 min each) and the signal was visualized with Immuno-Star™ WesternC™ Kit (BioRad) according to the manufacturer’s instructions. Band intensity was quantified with Quantity One software. Data were collected from three sham and three OVX hearts and each experiment was repeated two to three times.

**Data analyses.** Contraction amplitude (difference between systolic and diastolic length) was normalized to resting cell length. The time-to-peak contraction time was the time between initiation and peak shortening. The half-relaxation time was the time for 50% relaxation from peak contraction. Ca^2+^ and caffeine-induced transient amplitudes were the difference between systolic and diastolic Ca^2+^ concentrations. The rates of rise and 50% decay of depolarization-induced Ca^2+^ transients also were measured. Fractional SR Ca^2+^ release was the peak Ca^2+^ transient/the peak caffeine-induced transient. Ca^2+^ current was the difference between peak inward current and a reference point at the end of the voltage step. Ca^2+^ currents were normalized to cell capacitance. A more detailed analysis of the Ca^2+^ currents was carried out for currents elicited by test steps to 0 mV. Specifically, Ca^2+^ currents were integrated and the time constant (tau) for inactivation of the Ca^2+^ current was calculated from a single exponential function fitted to the decay phase of the current recordings. Gain was calculated as a ratio of peak Ca^2+^ transient/Ca^2+^ current.

**Statistics.** Statistical analyses were performed with SigmaStat (version 3.1, Systat Software, Inc.). Differences between means were evaluated by either a t-test or two-way repeated-measures analysis of variance (post hoc test=Student-Newman-Keuls). Graphs were constructed with Sigmaplot (version 8.0, Systat Software, Inc). Data are presented as means ± SEM and differences were considered significant if p<0.05.

**Chemicals.** Lidocaine, HEPES, EGTA, MgCl_2_, anhydrous DMSO, 4-aminopyridine and caffeine were purchased from Sigma-Aldrich Canada Ltd. (Oakville, ON, Canada). Fura-2 AM, was obtained from Invitrogen Inc. (Burlington, ON, Canada). All other chemicals were purchased from BDH Inc (Toronto, ON, Canada). Stock solutions of Fura-2 AM were prepared in anhydrous DMSO, with a final concentration of 0.2% DMSO, and stored at -20˚C until use. All other chemicals were dissolved in deionized water.

**REFERENCES**

1. Grandy SA, Howlett SE (2006) Cardiac excitation-contraction coupling is altered in myocytes from aged male mice but not in cells from aged female mice. Am J Physiol Heart Circ Physiol 291: H2362-2370.

2. Ferrier GR, Redondo IM, Mason CA, Mapplebeck C, Howlett SE (2000) Regulation of contraction and relaxation by membrane potential in cardiac ventricular myocytes. American journal of physiology Heart and circulatory physiology 278: H1618-1626.

3. Shutt RH, Howlett SE (2008) Hypothermia increases the gain of excitation-contraction coupling in guinea pig ventricular myocytes. American journal of physiology Cell physiology 295: C692-700.

4. Farrell SR, Ross JL, Howlett SE (2010) Sex differences in mechanisms of cardiac excitation-contraction coupling in rat ventricular myocytes. American journal of physiology Heart and circulatory physiology 299: H36-45.

5. Howlett SE (2010) Age-associated changes in excitation-contraction coupling are more prominent in ventricular myocytes from male rats than in myocytes from female rats. American journal of physiology Heart and circulatory physiology 298: H659-670.

6. Ferrier GR, Howlett SE (1995) Contractions in guinea-pig ventricular myocytes triggered by a calcium-release mechanism separate from Na+ and L-currents. The Journal of physiology 484 ( Pt 1): 107-122.

7. Delbridge LM, Bassani JW, Bers DM (1996) Steady-state twitch Ca2+ fluxes and cytosolic Ca2+ buffering in rabbit ventricular myocytes. The American journal of physiology 270: C192-199.

8. Katoh H, Schlotthauer K, Bers DM (2000) Transmission of information from cardiac dihydropyridine receptor to ryanodine receptor: evidence from BayK 8644 effects on resting Ca(2+) sparks. Circulation research 87: 106-111.

9. Picht E, Zima AV, Blatter LA, Bers DM (2007) SparkMaster: automated calcium spark analysis with ImageJ. American journal of physiology Cell physiology 293: C1073-1081.

10. Yang F, Aiello DL, Pyle WG (2008) Cardiac myofilament regulation by protein phosphatase type 1alpha and CapZ. Biochem Cell Biol 86: 70-78.

11. Patton C, Thompson S, Epel D (2004) Some precautions in using chelators to buffer metals in biological solutions. Cell Calcium 35: 427-431.

12. Bjornstad JL, Skrbic B, Marstein HS, Hasic A, Sjaastad I, et al. (2012) Inhibition of SMAD2 phosphorylation preserves cardiac function during pressure overload. Cardiovascular research 93: 100-110.

13. Cheng Y, Li W, McElfresh TA, Chen X, Berthiaume JM, et al. (2011) Changes in myofilament proteins, but not Ca(2)(+) regulation, are associated with a high-fat diet-induced improvement in contractile function in heart failure. American journal of physiology Heart and circulatory physiology 301: H1438-1446.

14. Rose RA, Sellan M, Simpson JA, Izaddoustdar F, Cifelli C, et al. (2011) Iron overload decreases CaV1.3-dependent L-type Ca2+ currents leading to bradycardia, altered electrical conduction, and atrial fibrillation. Circulation Arrhythmia and electrophysiology 4: 733-742.

15. Aldridge GM, Podrebarac DM, Greenough WT, Weiler IJ (2008) The use of total protein stains as loading controls: an alternative to high-abundance single-protein controls in semi-quantitative immunoblotting. Journal of neuroscience methods 172: 250-254.
